# Integrated Lipidomics and Flavoromics Analyses Reveal the Flavor Differences Between Breast and Leg Muscles of Xichuan Black-Boned Chicken

**DOI:** 10.3390/ani16071015

**Published:** 2026-03-26

**Authors:** Li Zhou, Wenfei Dong, Luyu Yang, Zhiyuan Zhang, Fumin He, Ruilong Xu, Chenkang Li, Xiangtao Kang, Donghua Li

**Affiliations:** College of Animal Science and Technology, Henan Agricultural University, Zhengzhou 450046, China; zhouli341125@126.com (L.Z.); 15103827042@163.com (W.D.); yly24580@163.com (L.Y.); zhang90392023@163.com (Z.Z.); 18736585267@163.com (C.L.); kangxiangtao@henau.edu.cn (X.K.)

**Keywords:** chicken, meat quality, lipidomics, flavoromics, flavor compounds, anatomical location

## Abstract

Xichuan black-boned chicken is a premium Chinese local breed in Xichuan County, Henan Province, China. However, the flavor characteristics of Xichuan black-boned chicken meat have not been systematically studied. Lipidomics and flavoromics approaches were used to analyze DLMs (differential lipid molecules) and DFCs (differential flavor compounds) in breast muscle (BM, *n* = 6) and leg muscle (LM, *n* = 6) of black-boned chicken, to reveal molecular mechanisms affecting meat quality in chicken. Lipidomics analysis reveals that 354 differential lipids are the differential abundance between the two groups, of which 33 are up-regulated and 321 are down-regulated in the BM group. These differential lipids were mostly enriched in glycerolipid metabolism, glycerophospholipid metabolism, and metabolic pathways. Flavoromics results demonstrate that there are 70 differential flavors between the two groups. Of these flavors, 59 are down-regulated and 11 are up-regulated in the BM group. These differential flavor compounds are mainly enriched in insect hormone biosynthesis and terpenoid backbone biosynthesis. Integrated lipidomics and flavoromics analysis shows that TG-type lipids and dodecanenitrile flavors may be the major related pairs. These findings not only enhance the understanding of the mechanism of chicken meat flavor formation but also provide novel perspectives for the improvement of meat quality.

## Simple Summary

Xichuan black-boned chicken is a well-known indigenous chicken breed in China. We evaluated the breast and leg muscles flavor profiles of the Xichuan black-boned chicken using lipidomics and flavoromics. A total of 354 differential lipid molecules and 70 differential flavor compounds were identified, of which TG-type lipids and dodecanenitrile may be the important associated pair at the two-omics levels. Collectively, our study provides valuable insights for further elucidating flavor formation mechanisms in chicken.

## 1. Introduction

Chicken meat is highly valued for its unique flavor characteristics [1]. In recent decades, researchers have primarily concentrated on the growth performance, reproductive performance and feed conversion ratio of chickens, and the growth rate and muscle yield of chicken have been significantly improved [2]. Nevertheless, chicken meat quality has deteriorated substantially with the increased growth rate [3]. Therefore, enhancing chicken meat quality while maintaining a high growth rate continues to present a considerable challenge. In recent years, improving the meat quality of chicken has become an important direction that many poultry breeders have been working hard on [4]. Meat quality is affected by many factors, including breed, age, anatomical location, and feeding environment [5]. Anatomical location is one of the key factors affecting chicken meat quality [6]. Currently, more and more scientists are acknowledging anatomical location as an important indicator of meat quality. Previous research has indicated that chicken muscles from different anatomical sites exhibit different quality traits, driven by differences in their tissue composition [7]. It is well known that breast and leg muscles are widely used as typical white and red muscles in chicken meat quality [8]. Therefore, in-depth research on the differences in chicken quality across different muscle parts and understanding of their molecular regulatory mechanisms are essential for improving chicken quality.

Recently, an increasing number of studies have aimed at the improvement of chicken meat quality [9]. As the demand for high-quality chicken continues to grow, the utilization of genetic resources in breeding programs has become a key strategy for improving meat quality [10]. Flavor is a crucial factor influencing consumers’ assessment of chicken meat quality [11]. However, the molecular mechanisms underlying these superior quality traits of chicken meat remain unclear. In recent years, the rapid development of multi-omics approaches, such as flavoromics and lipidomics, have offered novel insights for in-depth exploration of the mechanisms underlying chicken meat flavor formation [12]. Lipidomics, a rapidly developing important branch of metabolomics, enables a comprehensive understanding of lipid components in biological systems [13]. Flavoromics offers a new direction for associating the differential molecular expression patterns with flavor substances [14]. Overall, the combination of lipidomics and flavoromics of chicken could be a complementary analytical strategy for comprehensive analysis. Unfortunately, there is currently a lack of research on multi-omics strategies in chickens for evaluating meat quality, especially focusing on muscles from different anatomical parts. Recently, integrated multi-omics technologies have been widely recognized as a powerful tool for studying meat quality [15]. There is enormous development potential in Chinese native local chicken breeds, which have a lower growth rate and feed conversion efficiency but are excellent in meat quality and flavor [16]. The Xichuan black-boned chicken is known for its healthy meat, low fat, high-protein content, and a unique flavor that are deeply favored by consumers [17]. In 2010, it was officially recognized as an essential breed for resource conservation by the Ministry of Agriculture of China. In this context, Xichuan black-boned chickens, as a rare breed of local chickens in China, play an important role in meeting the market demand [18]. However, no studies have reported the integration of lipidomics and flavoromics techniques to assess the meat quality of black-bone chicken.

In this study, flavoromics and lipidomics strategies were comprehensively applied to systematically compare and examine the differences in critical flavor characteristics and lipid metabolites between the breast and leg muscles of Xichuan black-boned chickens. Furthermore, Pearson′s correlation analysis was used to investigate the association between critical lipids and aroma compounds in chicken meat. This study provides new perspectives into molecular mechanisms underlying flavor formation of local chicken breeds such as the black-boned chicken, and lays a theoretical foundation for the breeding of premium chicken varieties.

## 2. Materials and Methods

### 2.1. Animals and Samples Acquisition

Six 365-day-old female Xichuan black-boned chickens used in the present study were obtained from the Poultry Breeding Farm of Henan Agricultural University (Xinxiang, China). All hens were raised under similar feeding conditions and fed the same diet. All experimental procedures were approved by the Animal Policy and Welfare Committee of Henan Agricultural University (No. S20190196). Samples of muscle tissue from the right *pectoralis major* (BM, *n* = 6) and right *iliotibialis* (LM, *n* = 6) were taken from healthy Xichuan black-boned chickens after euthanasia. All muscle samples were rapidly frozen in liquid nitrogen for further processing.

### 2.2. Targeted Lipidomics

#### 2.2.1. Lipid Separations of Samples

Lipid separations were performed according to Pinto et al. with some modifications [19]. From the fresh pectoralis major (*n* = 6) and right leg muscle *(n* = 6) obtained, approximately 20 mg of each sample was isolated for lipid extraction. A 20 mg sample was put in an Eppendorf tube, and chloroform and methanol (2:1, *v*/*v*) were added in sequentially. The mixture was pre-cooled at −20 °C for 5 min with two steel balls placed in the tube, followed by grinding using an MM400 ball mill (Retsch, Haan, Germany). The dissolved samples were then thoroughly mixed for a duration of 2 min using a MIX-200 multitube vortex mixer (Tuohe Technology, Shanghai, China). Next, the sample was extracted with a KQ5200E ultrasonic instrument (Kunshan Shumei Ultrasonic Instrument Co., Ltd., Kunshan, China) for 15 min under ice-water cooling. After allowing it to solidify for 1 h, 200 μL of ultra-pure H_2_O was added, followed by vortexing for 1 min. After centrifugation at 12,000 rpm for 10 min at 4 °C (5424R, Eppendorf, Hamburg, Germany), 300 μL of the organic layer was transferred into a new tube. The prepared samples were subjected to vacuum evaporation until dryness. The dried powder was mixed with 200 μL of lipid reconstitution solution. The supernatant was filtered through a 0.22 μm membrane to obtain a filtrate.

#### 2.2.2. Liquid Chromatography-Mass Spectrometry

A quality control (QC) sample was prepared by mixing an equal volume (20 µL) of extract from each meat sample. To monitor and evaluate the stability and robustness during lipid detection, a QC sample was analyzed after every six meat samples throughout the instrumental run. The lipids were analyzed using Ultra Performance Liquid Chromatography (ExionLC^TM^ AD, Framingham, MA, USA) and Mass Spectrometry (QTRAP^®^ 6500+, Pisa, Italy). A C30 column (Waters, Waltham, MA, USA) was applied for chromatographic separation and maintained at 45 °C. For lipidomics analysis, mobile phases A and B consisted of acetonitrile/water (60:40, *v*/*v*) and acetonitrile/isopropanol (10:90, *v*/*v*), respectively, both supplemented with 0.1% formic acid. The samples were injected from the autosampler, and each injection volume was 2 μL. The gradient was programmed as follows: A/B was 80:20 (*v*/*v*) at 0 min, 70:30 (*v*/*v*) at 2 min, 40:60 (*v*/*v*) at 4 min, 15:85 (*v*/*v*) at 9 min, 10:90 (*v*/*v*) at 14 min, 5:95 at 15.5 min (*v*/*v*), 5:95 at 17.3 min (*v*/*v*), 80:20 (*v*/*v*) at 17.5 min, and 80:20 (*v*/*v*) at 20 min.

The mass spectrometry conditions were as follows: electrospray ionization temperature—500 °C; spray voltage −5.5 kV for positive mode and −4.5 kV for negative mode; ion source gas 1 (GS1)—45 psi; normalized collision energy—30 eV; ion source gas 2 (GS2)—55 psi.

#### 2.2.3. Data Processing and Analysis of Lipidomics

The LipidSearch software (Version 4.1.3) was applied to recognize lipid molecules, lipid fragment peaks, extract peaks, and identify lipids. Lipids were clearly distinguished based on retention time (RT) and mass to charge ratio (*m*/*z*) and were quantified by normalization intensity of the ions to internal standards. Analyst 1.6.3 software was employed for the processing of mass spectral data. Qualitative analysis of lipids in the meat samples was performed by mass spectrometry based on the MWDB database. The qualitative analysis of target substances was performed based on their precursor ions, characteristic fragment ions, and RT. The peak areas of all chromatographic peaks were integrated. The MultiQuant software (Version 3.3) was utilized to correct the chromatographic peaks of each substance detected in different meat samples, ensuring the accuracy of relative quantification. Principal component analysis (PCA) and orthogonal partial least squares discriminant analysis (OPLS-DA) were employed for multivariate statistical analysis. Cross-validation of the established PLS-DA model was estimated according to the calculated Q^2^, R^2^X, and R^2^Y values. Hierarchical cluster analysis and correlation analysis were performed with R software (Version 3.5.1, Version 2.7.1). The screened lipids with Variable Importance in Projection (VIP) scores > 1 and *p*-values < 0.05 in the statistical difference analysis were considered differential lipid molecules (DLMs). These lipid molecules were functionally enriched by mapping to the Kyoto Encyclopedia of Genes and Genomes (KEGG) signaling pathways, revealing their involvement in specific biological processes.

### 2.3. Untargeted Flavoromics

#### 2.3.1. Flavor Compounds Extraction of Samples

Flavor compounds were extracted from goat *longissimus dorsi* muscle as previously described by Li et al. [20], with some modifications. For the flavor compounds extracted, the *pectoralis major* samples (BM) were obtained from the same location at the breast muscle (*n* = 6), and the right leg muscles (LM) were obtained from the same location of the *iliotibial* (*n* = 6). Chicken meat samples were taken out of storage and thawed at 4 °C. The samples were incubated with shaking at 60 °C for 5 min, and volatile compounds were extracted by a solid-phase microextraction fiber (Supelco, Bellefonte, PA, USA) at the same temperature for 15 min, and subsequently desorbed in the Gas Chromatography (GC) injector at 250 °C for 5 min. Each sample was analyzed in triplicate for GC–MS/MS analysis.

#### 2.3.2. Chromatography and Mass Spectrometry Conditions

Subsequently, all flavor compounds of equal volume were combined to prepare QC samples. During instrumental analysis, one QC sample was inserted in every six samples to evaluate the repeatability of the entire analytical procedure. GC-MS/MS analysis was performed using an 8890 GC apparatus (Agilent, Santa Clara, CA, USA) and a 7000D MS chromatograph (Agilent, Santa Clara, CA, USA). High-purity nitrogen (>99.9999%) was employed as the carrier gas. A constant flow rate of 1.2 mL/min was used throughout the analysis. The chromatographic column was DB-5MS (Agilent, Santa Clara, CA, USA). All samples were prepared and analyzed in triplicate. Chromatographic conditions: initial temperature of 40 °C, raised to 100 °C at 10 °C/min, then to 180 °C at 7 °C/min, and finally to 280 °C at 25 °C/min, followed by a 5 min hold.

The mass spectrometry conditions were as follows: electron bombardment ionization source with 70 eV electron energy, ion source temperature was kept at 230 °C, quadrupole mass detector temperature was set to 150 °C, transfer line temperature maintained at 280 °C. The data were obtained through data-dependent acquisition by mass spectrometry.

#### 2.3.3. Data Processing and Analysis of Flavoromics

Raw data were processed using MassHunter (Version 8.0) software. Identification of flavor compounds was performed by matching mass spectra and experimental retention indices to the NIST 2017 database (Gaithersburg, MD, USA). The relative content of each flavor component was determined by peak area total sum normalization. Pearson′s correlation analysis was performed on QC samples to assess data reliability. Multivariate statistical analyses including PCA and OPLS-DA were used to evaluate flavoromics data. The leave-one-out cross-validation was used to assess the robustness of the model. The model’s VIP was determined by multiple cross-validation. Differential flavor compounds (DFCs) were identified according to the criteria: fold change ≥ 1.5 or fold change ≤ 0.67 and VIP > 1. KEGG functional enrichment analysis was conducted to explore the biological significance of flavor compound variations.

### 2.4. Correlation Analysis Between Lipidomics and Flavoromics

Spearman′s correlation analysis was performed to analyze the correlation between differential flavor compounds and lipids. Statistical significance was defined as *p* < 0.05. Matrix diagram analysis was performed and visualized using the R package. Furthermore, their interactive relationships were visualized by constructing correlation networks with |r| > 0.5.

### 2.5. Data Statistical Analysis

All statistical analyses were performed using SPSS 26 software, and one-way analysis of variance was employed for sample comparisons. Significance testing between groups was conducted using Student′s *t*-test. Data were shown as mean ± standard deviation. Significant differences were identified as *p* < 0.05. Other figures were plotted using Prism 8 software (GraphPad).

## 3. Results

### 3.1. Lipidomics Analysis

**Figure 1:**
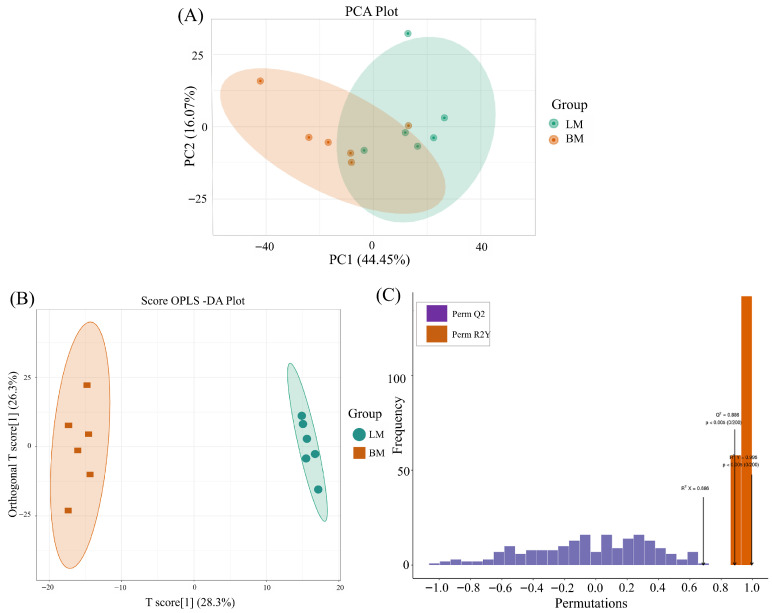
Multivariate statistical analysis. (**A**) Principal component analysis (PCA) of muscle lipids in chickens. Yellow represents BM group; green represents LM group. PC1, PC2 represent principal component 1, principal component 2, respectively. (**B**) Orthogonal projections to latent structures discriminant analysis (OPLS-DA) based on lipidomics data from breast and leg muscles. Yellow represents BM group; green represents LM group. (**C**) OPLS-DA validation plots based on lipidomics data from breast and leg muscles (R^2^X = 0.686, R^2^Y = 0.995, Q^2^ = 0.886).

To elucidate the differences in lipid composition of muscle in Xichuan black-boned chickens, lipidomics analysis was conducted on the breast and leg muscles, followed by data normalization. Lipidomics analysis of the 12 samples was performed; 967 lipids were identified. PCA of the lipidomic data from the LM vs. BM comparison exhibited a partial separation, indicating small differences between lipid profiles (Figure 1A). Subsequently, the results of the PCA-3D study are presented in Figure S1. The OPLS-DA shows that there are clear differences between breast and leg muscles (Figure 1B). The corresponding OPLS-DA validation plots show satisfactory R^2^X (0.686), R^2^Y (0.995), and Q^2^ (0.886) scores (Figure 1C). This reveals that the model′s predictive performance is highly significant and not due to random variation, highlighting the reliability and validity of the model.

**Figure 2:**
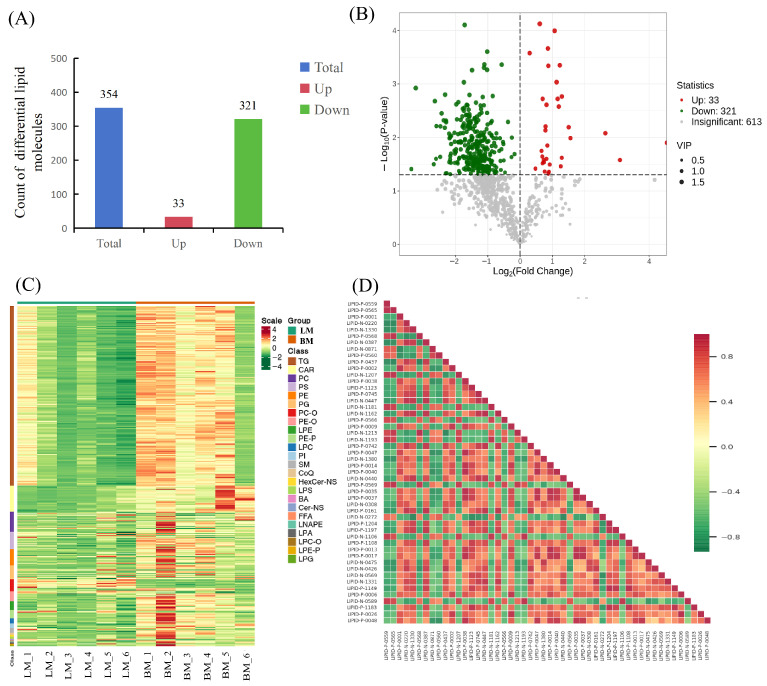
Differential lipid molecules in breast and leg muscles. (**A**) The number of differential lipid molecules in LM vs. BM. (**B**) Volcano plot of differential lipids in breast and leg muscles. Red dots represent up-regulated lipid molecules, green dots represent down-regulated lipid molecules, and gray dots coordinate non-differential lipid molecules. (**C**) Hierarchical clustering analysis of breast and leg muscles by candidate lipid molecules. Red shows high abundance, and green shows low abundance. (**D**) Correlation heatmap of differential lipids. Horizontal axis represents differential lipid name, and vertical axis represents differential lipid name. Red and green denote strong positive and negative correlation, respectively.

In total, 354 lipid molecules were identified as significantly different between LM and BM by setting VIP > 1 and *p* < 0.05 (Figure 2A and Supplementary Materials, Table S1). Among these, 33 lipid molecules were up-regulated in BM, while 321 lipid molecules were down-regulated. The results are depicted in a volcano plot (Figure 2B). A heatmap of the differential lipid molecules demonstrates that the samples exhibited good clustering performance. After performing clustering analysis, the lipid molecules were categorized into two clusters that exhibited differential expression patterns between breast and leg muscles (Figure 2C). To further explore the potential relationship between the differential lipids, a correlation heat map was established using R packages. The top 50 lipid molecules with the highest VIP scores were selected from the diagram (Figure 2D).

**Figure 3:**
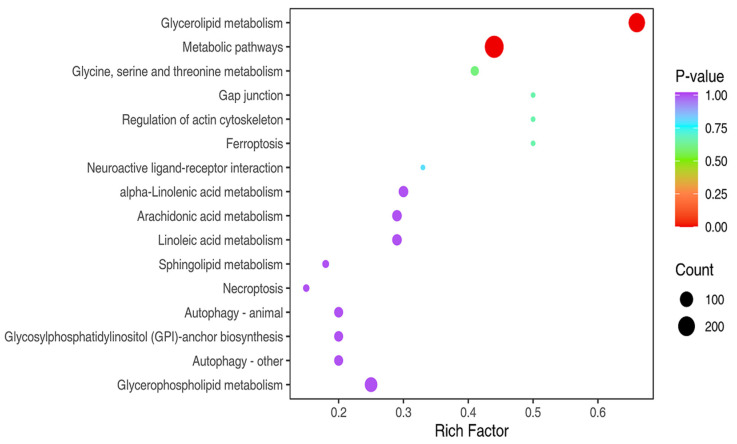
KEGG functional annotation of differential lipid molecules. The horizontal axis denotes the rich factor, and the vertical axis represents the name of the signaling pathway. The size of circle represents the quantity of differential lipid molecules involved in this pathway. The color of circle corresponds to distinct *p*-value ranges.

For the interpretation of lipidomics data, pathway enrichment analysis was carried out on differential lipid molecules via the KEGG. The results showed that glycerolipid metabolism, glycerophospholipid metabolism, and metabolic pathways were the most impacted pathways (Figure 3). From the above results, it was inferred that these signaling pathways might constitute potential pathways associated with chicken meat flavor in the LM vs. BM groups.

### 3.2. Flavoromics Analysis

**Figure 4:**
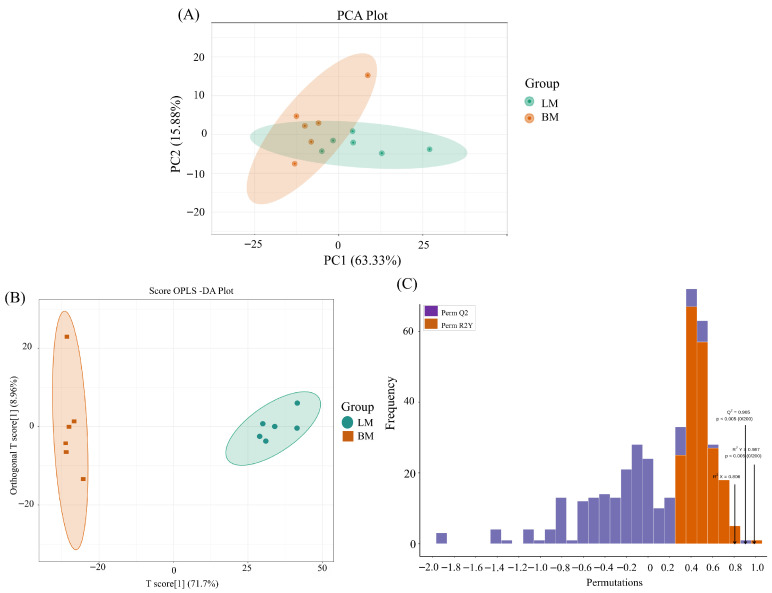
(**A**) Principal component analysis (PCA) of muscle flavor compounds in chickens. Yellow points represent BM group; green points represent LM group. PC1, PC2 represent principal component 1, principal component 2, respectively. (**B**) Orthogonal partial least squares discriminant analysis (OPLS-DA) based on flavoromics data from breast and leg muscles. Yellow points show BM group; green points show LM group. (**C**) Orthogonal partial least squares discriminant analysis (OPLS-DA) validation plot based on flavoromic data from breast and leg muscles (R^2^X = 0.806, R^2^Y = 0.987, Q^2^ = 0.905).

Furthermore, a flavoromic strategy was employed to screen for volatile compounds that showed notable variations between the breast and leg muscles. A total of 232 flavor compounds were detected and identified in the present study. To assess differences in the two groups, PCA was employed. PCA showed distinct separation between two muscle types: PC1 accounted for 63.33% of the total variance, and PC2 contributed 15.88% (Figure 4A). PCA-3D of all samples demonstrates distinct clustering patterns between the two groups (Figure S2). The score plot of the OPLS-DA model indicates that comparisons between the LM and BM groups show distinct separation (Figure 4B). In the permutation test, plots of the OPLS-DA models yielded Q^2^ > 0.905, R^2^X = 0.806, and R^2^Y > 0.987, confirming the reliability of the established OPLS-DA model (Figure 4C).

**Figure 5:**
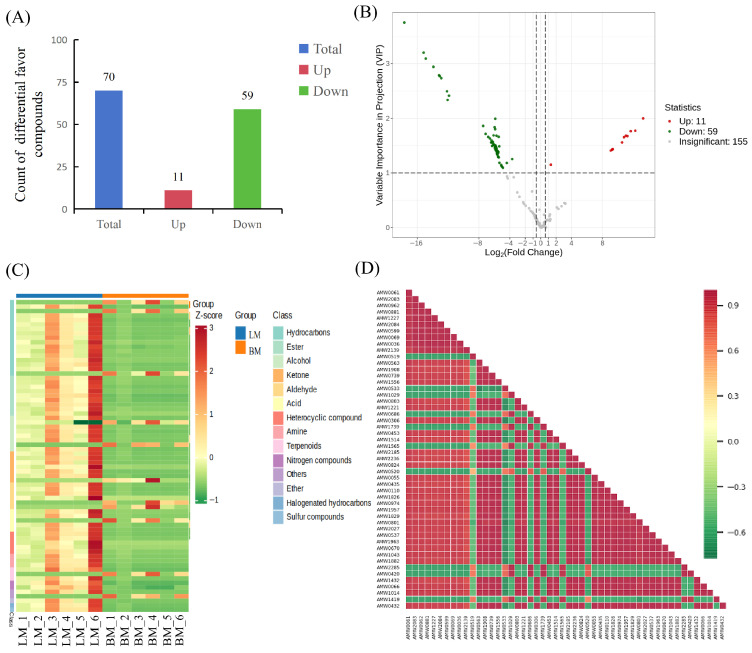
Analysis of differential flavor compounds in breast and leg muscles. (**A**) Bar chart for screening of differential flavor compounds. (**B**) Volcano plot for screening of differential flavor compounds. Green dots indicate the down-regulated flavor compounds, red dots indicate the up-regulated flavor compounds, and gray dots indicate the non-significant differential flavor compounds. (**C**) Hierarchical clustering analysis of breast and leg muscles by candidate flavor compounds. Red shows high abundance, and green indicates low abundance. (**D**) Correlation heatmap of differential flavor compounds. Horizontal axis indicates the names of differential flavor compounds and vertical axis indicates the names of differential flavor compounds. Red and green denote strong positive and negative correlation, respectively.

To investigate molecular variations between breast and leg muscles, significant differential flavor compounds were identified. Using the criteria of fold change ≥ 1.5 or fold change ≤ 0.67 and VIP > 1, we identified flavor compounds with significant differences between breast and leg muscles (Supplementary Materials, Table S2). Among these, 11 were found to be up-regulated, and 59 were down-regulated (Figure 5A). A volcano plot is constructed to visualize the up-regulated and down-regulated flavor compounds (Figure 5B). Meanwhile, in order to ensure the accuracy of the analysis, the screened differential flavor compounds were analyzed by hierarchical cluster analysis in which compounds with the same or similar expression patterns are clustered (Figure 5C). Next, we selected the top 50 differential flavor compounds with the largest VIP values and prepared a correlation heatmap (Figure 5D).

**Figure 6:**
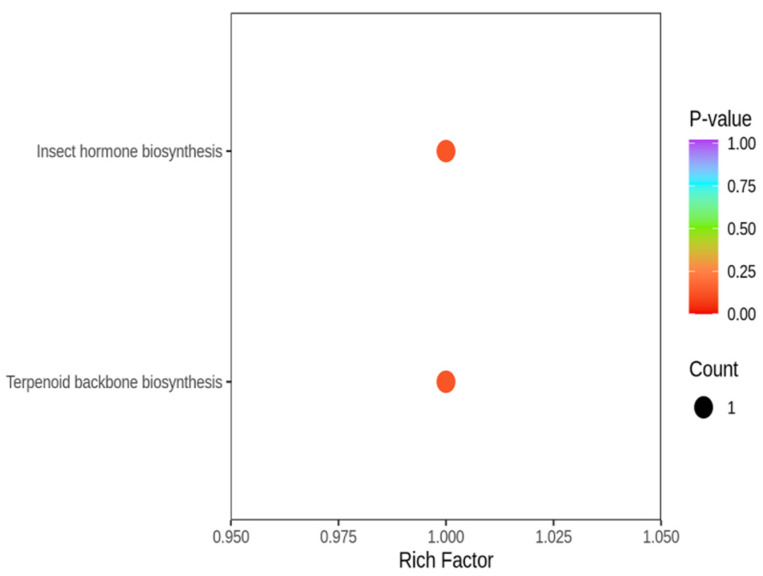
KEGG functional annotation of differential flavor compounds. X axis indicates rich factor of each signaling pathway, and Y axis shows signaling pathway name. Circle color reflects *p*-value and circle size represents the number of enriched differential flavor compounds.

Furthermore, KEGG functional enrichment analysis was conducted to identify the signaling pathways related to flavor formation (Figure 6). The differential flavor compounds were mainly enriched in metabolic signaling pathways related to insect hormone biosynthesis and terpenoid backbone biosynthesis, both of which are important for flavor formation. These findings demonstrate that the disparities in flavor formation between the two distinct muscle types may be ascribed to the differential expression of flavor compounds linked to these pathways.

### 3.3. Correlation Analysis Between Lipid Molecules and Flavor Compounds

**Figure 7:**
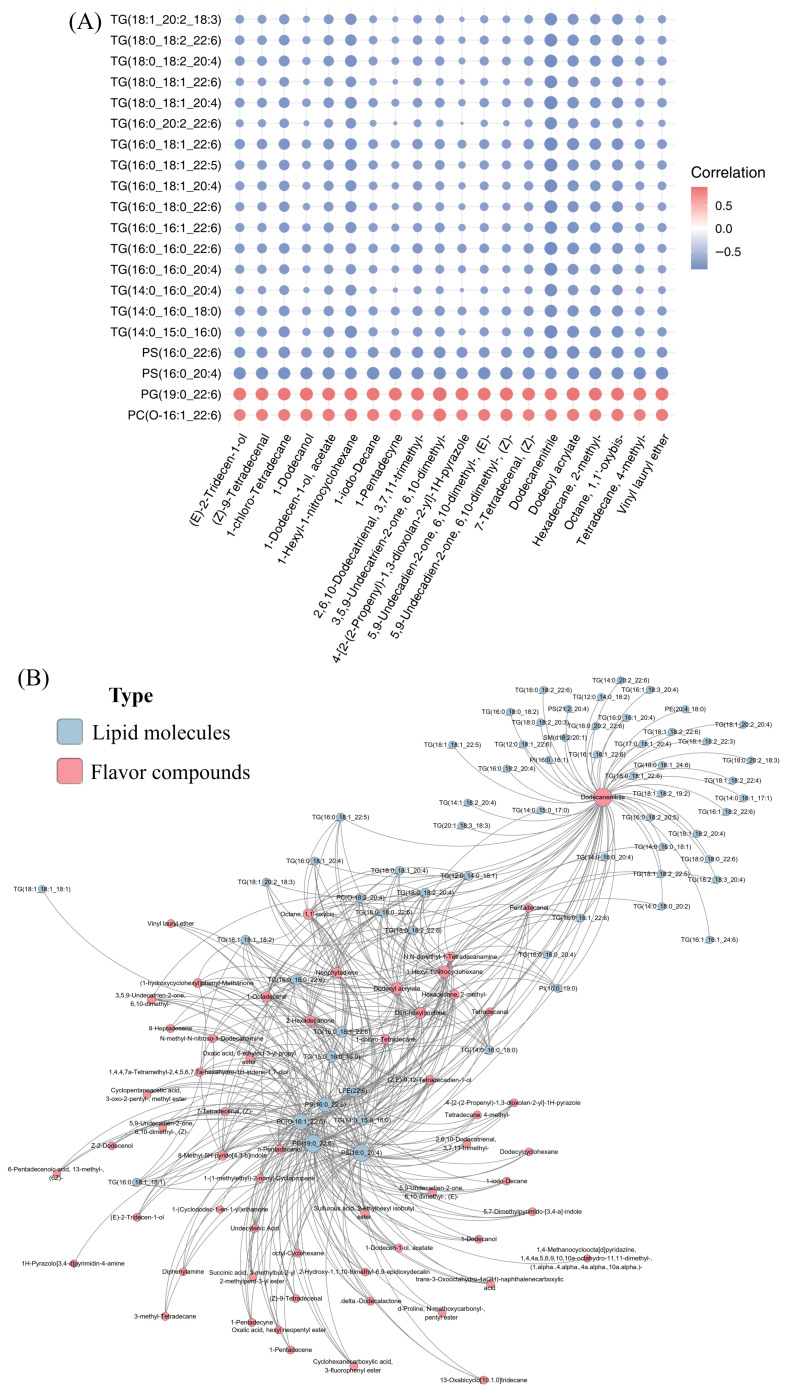
The results of multi-omics joint analysis. (**A**) Matrix diagram of the correlation between the top 20 differential lipid molecules and the top 20 differential flavor compounds. The horizontal axis indicates the names of differential lipid molecules and the vertical axis indicates the names of differential flavor compounds. Red denotes positive correlation, while blue denotes negative correlation. (**B**) Construction of correlation network among differential lipid molecules and differential flavor compounds. Red nodes indicate flavor compounds, while blue nodes indicate lipid molecules.

In order to further elucidate the regulation mechanism of chicken meat flavor, the correlation analysis of the top 20 differential lipid molecules and the top 20 differential flavor compounds were performed. The results of the Matrix diagram show that the top 20 screened lipid molecules are mostly TG-type, indicating that TG-type lipids are mainly responsible for regulating the molecular mechanism of breast and leg muscles to improve the meat quality of Xichuan black-boned chicken (Figure 7A). Meanwhile, we screened out the lipid–flavor pairs with the correlation coefficient |r| > 0.5 (*p* < 0.05), and then we performed network association, as shown in Figure 7B. The results show that flavor compounds of dodecanenitrile and lipid molecules of the TG type are relatively critical.

## 4. Discussion

Global per capita meat consumption continues to rise, especially for chicken [21]. Chicken meat is a nutrient-rich food that helps to maintain and improve human health [22]. In recent years, the research on chicken meat has attracted more and more attention [23]. Factors such as breed, diet, and age influence the flavor formation of chicken meat [24]. Nevertheless, the molecular mechanism underlying flavor variations in chicken meat from different anatomical locations remains unclear. This knowledge gap underscores the necessity of exploring the flavor characteristics of chicken meat from different anatomical locations. China possesses a long history of chicken farming, and its indigenous breeds provide exceptionally diverse genetic resources [25]. Native chicken breeds in China are highly valued for their distinctive flavor. The Xichuan black-boned chicken is a traditional local breed in China with a long history of breeding [26]. Owing to the limited relevant studies on the specific lipid composition and flavor profile of Xichuan black-boned chicken, it is challenging to establish practical grading standards for this breed. Furthermore, few studies have investigated the effects of different parts on metabolic pathways that may contribute to chicken flavor formation [27]. Therefore, studying the difference in lipids in the breast and leg muscles of Xichuan black-boned chickens may provide new insights for improving meat quality. Herein, we employed a lipidomics and flavoromics strategy integrated with UPLC-MS/MS and GC-MS/MS to analyze both lipid molecules and volatile compounds.

As a branch of metabolomics, lipidomics serves as a powerful tool for characterizing lipid molecules based on their unique properties [28]. Screening lipids associated with meat quality traits using this novel technique offers valuable insights into the potential regulatory mechanisms underlying meat quality development [29]. In the present study, lipidomics analysis was used to identify and evaluate variations in the lipid composition and distribution in the Xichuan black-boned chicken muscle from various anatomical locations. Here, we determined the lipid profiles of two muscle types in Xichuan black-boned chickens. Between the LM and BM, a total of 354 different lipids (33 up-regulated, 321 down-regulated) were found, with many involved in processes such as glycerolipid metabolism, glycerophospholipid metabolism, and metabolic pathways. Glycerolipid metabolism is crucial for cell lipid storage and cell membrane homeostasis [30]. A recent study reported that glycerophospholipid metabolism is one of the most crucial signaling pathways associated with lipid variations during poultry (goose) growth [31]. In addition, the metabolic pathway also plays an important role in adipose tissue development and lipid metabolism [32]. These different lipid molecules and signaling pathways may account for the variations in meat quality between the breast and leg muscles in Xichuan black-boned chickens.

The factors influencing chicken muscle flavor formation are highly complex and diverse [33]. A single omics technology is no longer adequate to clarify the complex features and interrelationships associated with flavor formation [34]. Notably, flavoromics has been widely used in the livestock and poultry industry [35]. To compare the differential flavor compounds of the breast and leg muscles in Xichuan black-boned chicken, a flavoromics analysis method based on GC-MS was used to examine their characteristics. There are 70 significant differential flavor compounds between the LM and BM groups. A total of 11 flavor compounds are up-regulated, while 59 are down-regulated. Among these odorants, hydrocarbons, aldehydes, esters, alcohols, and acids were the main components of chicken meat aroma. This finding agrees with previous studies [36]. The diversity of flavor compounds present in the different muscles of these chickens may account for their complex flavor profiles [37]. When comparing the flavor compound profiles of breast and leg muscles in Xichuan black-boned chicken, more flavor compounds were identified, with most of them downregulated in the leg muscles. KEGG pathway analysis shows that the differential flavor compounds are mainly enriched in insect hormone biosynthesis and terpenoid backbone biosynthesis. Insect hormone biosynthesis is primarily composed of the ecdysone and juvenile hormone [38]. It has been reported that ecdysone regulates fat body remodeling by triggering apoptosis, autophagy, and matrix metalloproteinase-dependent cell dissociation [39]. The juvenile hormone is primarily an acyclic sesquiterpenoid that can freely enter cells via diffusion due to its lipophilic nature [40]. Previous studies indicate that terpenoid backbone biosynthesis is a key signaling pathway for fat deposition in broiler chickens [41]. Therefore, the above pathways play a vital role in lipid metabolic process.

The integration of multi-omics data provides a promising strategy to elucidate molecular mechanisms underlying livestock and poultry meat quality [42,43]. Therefore, in order to investigate the differences in flavor compounds between distinct muscle types in chickens, this study further conducted a multi-omics integrative analysis. The main oxidation products from lipid molecules, namely lipid hydroperoxides and conjugated dienes, are further decomposed into secondary degradation products to generate a variety of volatile organic compounds, such as acids, ketones, furans, and heterocycles, causing diversity of volatile characteristics in meat quality [44]. We filtered out the top 20 DLMs with relatively high Pearson correlation coefficients |r| as key DLMs between the two groups. The correlation coefficient results show that the top 20 lipid molecules are mostly TG types. TG has been shown to be an important lipid molecule for binding and generating flavor compounds [45]. Liu’s research indicates that the highest types and contents of TG may play a key role in preserving the aroma of roasted mutton [46]. Furthermore, the correlation network between different lipids and key volatile flavor substances was established. The results of the correlation showed that dodecanenitrile and TG-type lipids were strongly correlated, implying that they may have potential important metabolic functions in the process of meat quality formation. This is similar to latest findings on other meat [47,48]. In summary, the regulatory network between lipid molecules and flavor compounds provides a theoretical basis for genetic improvement of meat quality in poultry.

There are some limitations in the present research. It is very inadequate to draw a reliable conclusion with a small number of breast muscle and leg muscle samples in Xichuan black-boned chicken, and more samples are needed for further validation. In addition, the potential relationship between TG-type lipid changes and dodecanenitrile substances needs further research. In summary, our data highlights the correlation patterns and key lipid–flavor interactions underlying omics variations between breast and leg muscles of Xichuan black-boned chickens.

## 5. Conclusions

The present study is the first to systematically analyze lipid and volatile compound profiles in breast and leg muscles of Xichuan black-boned chickens. A total of 354 lipid molecules and 70 flavor compounds differentiate between breast and leg muscles. Moreover, our findings provide important insights into the molecular regulatory mechanism underlying flavor differences between breast and leg muscles, and offer valuable references for the exploitation, utilization and genetic breeding of high-quality broiler breeds.

## Data Availability

All data included in this work are available upon request by contact with the corresponding author.

## Supplementary Materials

The following supporting information can be downloaded at: https://www.mdpi.com/article/10.3390/ani16071015/s1. Figure S1: PCA-3D of the lipidomics data from the LM vs. BM; Figure S2: PCA-3D of the flavoromics data from the LM vs. BM; Table S1: Identification of differential lipid molecules; Table S2: Identification of differential flavor compounds.
