# Supplementary figures and images for: Integrated Lipidomics and Flavoromics Analyses Reveal the Flavor Differences Between Breast and Leg Muscles of Xichuan Black-Boned Chicken
Source: Animals (Basel). 2026 Mar 26;16(7):1015. doi: 10.3390/ani16071015 (PMC13072344; doi:10.3390/ani16071015)

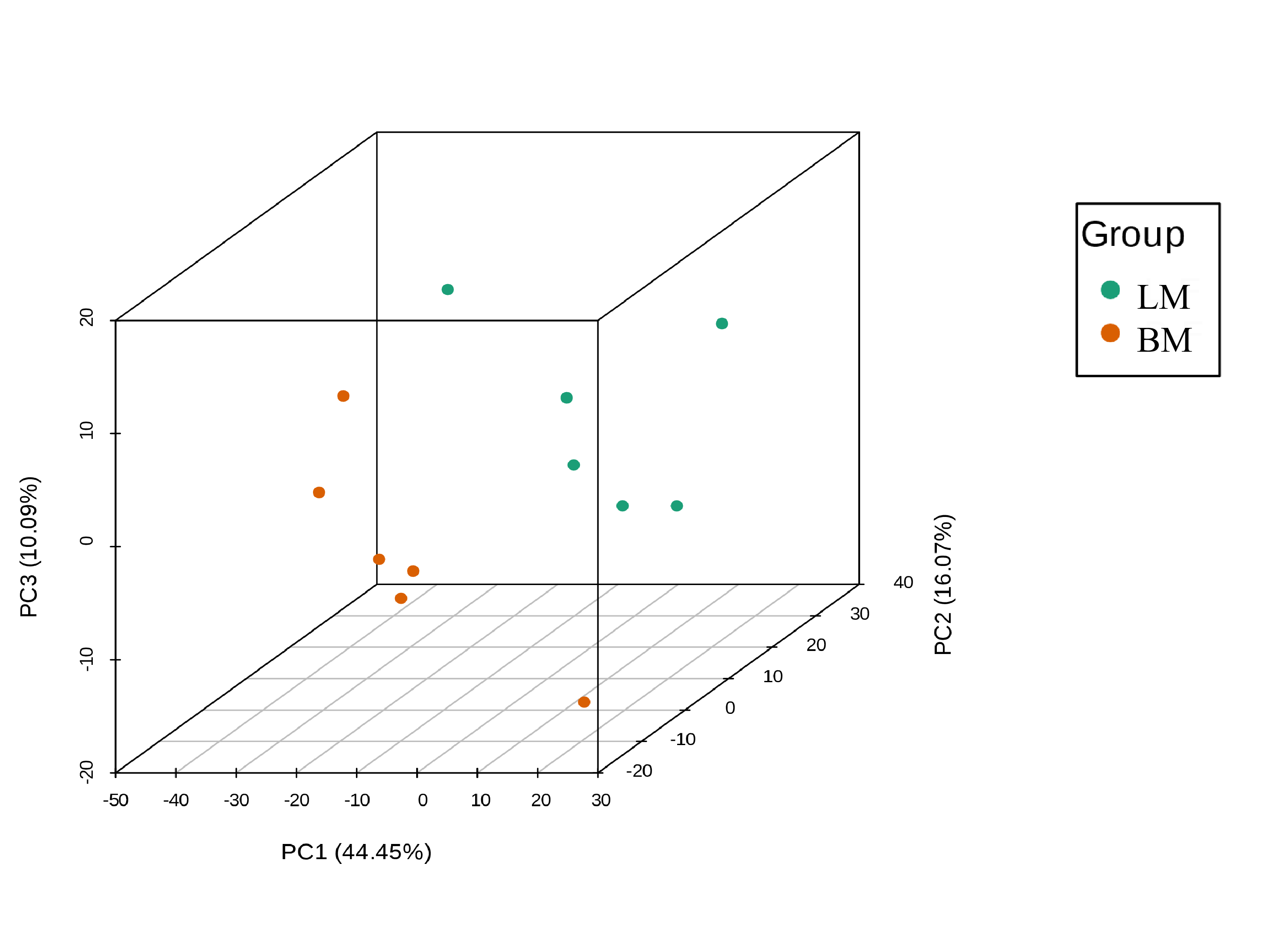

Supplement: Supplementary file 1 [file animals-16-01015-s001.zip › Figure S1.jpg]

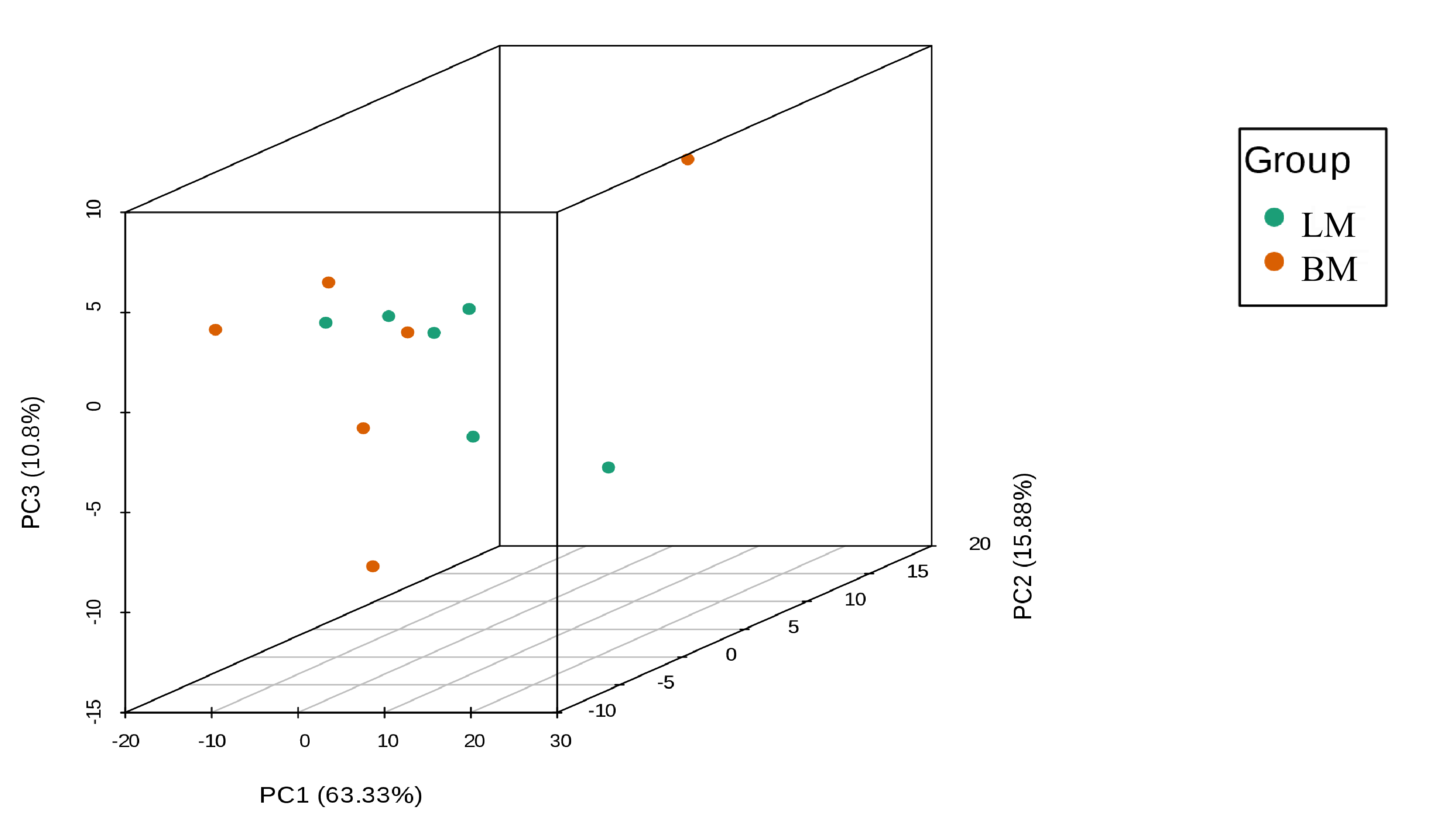

Supplement: Supplementary file 1 [file animals-16-01015-s001.zip › Figure S2 .jpg]
